# Directed evolution of the aryl-alcohol oxidase: Beyond the lab bench

**DOI:** 10.1016/j.csbj.2020.06.037

**Published:** 2020-06-29

**Authors:** Javier Viña-Gonzalez, Miguel Alcalde

**Affiliations:** Department of Biocatalysis, Institute of Catalysis, CSIC, Cantoblanco, 28049 Madrid, Spain

**Keywords:** Directed evolution, Aryl-alcohol oxidase, *Saccharomyces cerevisiae*, Chimeric signal peptide, Aromatic secondary alcohol, 2,5-furandicarboxylic acid

## Abstract

Aryl-alcohol oxidase (AAO) is a fungal GMC flavoprotein secreted by white-rot fungi that supplies H_2_O_2_ to the ligninolytic consortium. This enzyme can oxidize a wide array of aromatic alcohols in a highly enantioselective manner, an important trait in organic synthesis. The best strategy to adapt AAO to industrial needs is to engineer its properties by directed evolution, aided by computational analysis. The aim of this review is to describe the strategies and challenges we faced when undertaking laboratory evolution of AAO. After a comprehensive introduction into the structure of AAO, its function and potential applications, the different directed evolution enterprises designed to express the enzyme in an active and soluble form in yeast are described, as well as those to unlock new activities involving the oxidation of secondary aromatic alcohols and the synthesis of furandicarboxylic acids.

## Graphical abstract

**Figure:**
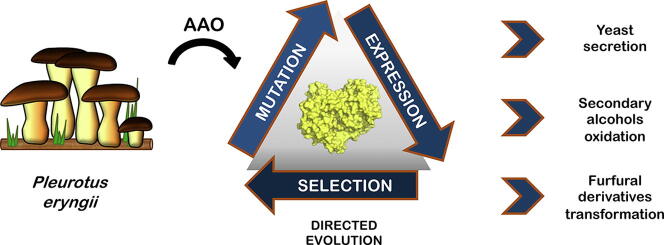

## 1 Introduction

### 1.1 Natural role and substrate scope

**Fig. 1:**
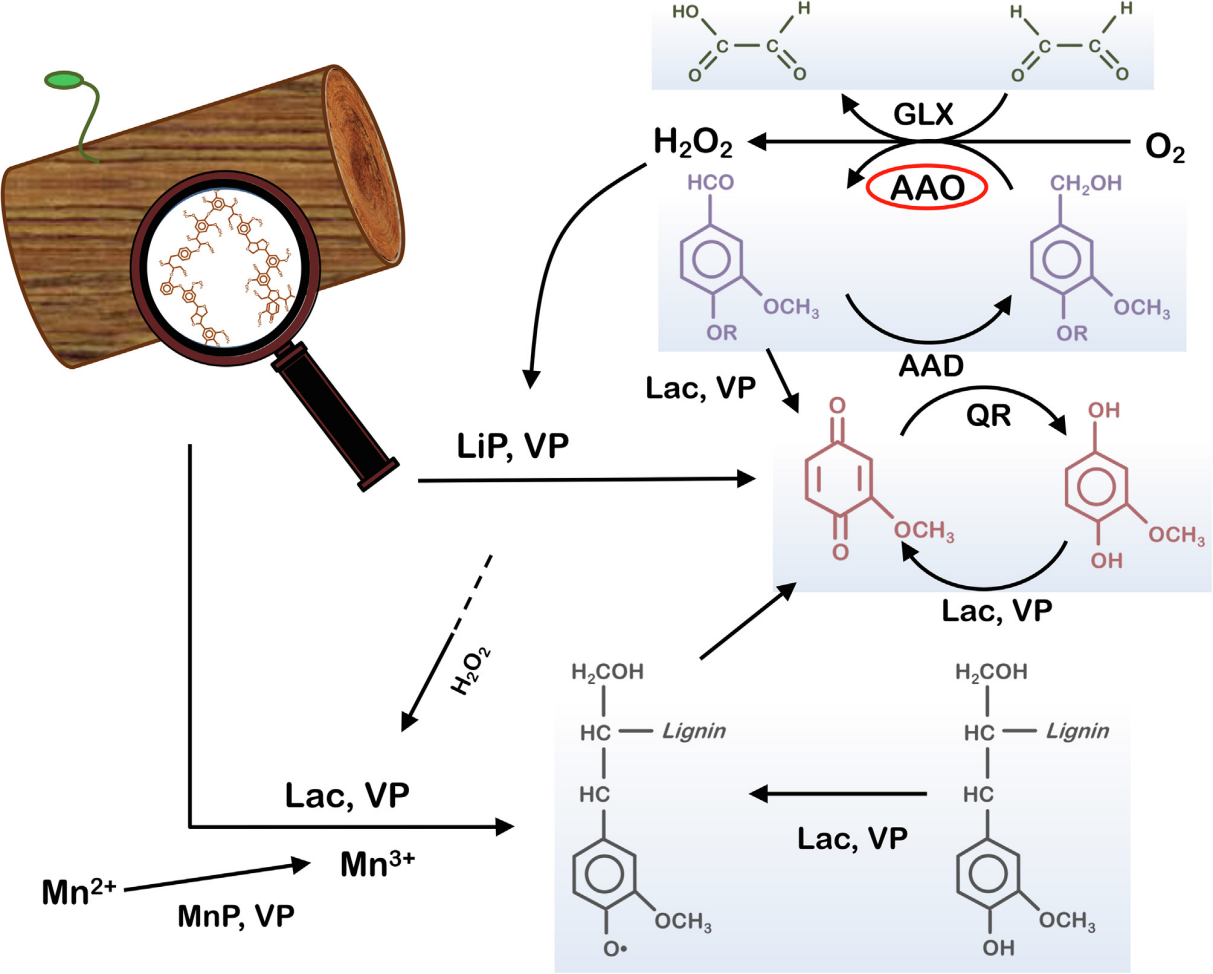
Consortium of ligninases: Lac, laccase; LiP, lignin peroxidase; MnP, manganese peroxidase; VP, versatile peroxidase; GLX, glyoxal oxidase; AAO, aryl-alcohol oxidase; AAD, aryl-alcohol dehydrogenase; QR, quinone reductases.

Adapted from [9].

Aryl-alcohol oxidase (AAO, EC 1.1.3.7) is an extracellular fungal flavoenzyme that supplies H_2_O_2_ to peroxidases and peroxygenases during natural lignin degradation. Lignin represents around 10–25% of lignocellulose, the most abundant biological feedstock in nature, and it is a recalcitrant three-dimensional aromatic biopolymer that only a few organisms can degrade, mainly white-rot fungi [1], [2], [3]. Coordinated fungal attack on lignin by white-rot basidiomycetes is mediated by an enzymatic consortium of ligninolytic oxidoreductases (commonly referred to as ligninases), which include high-redox potential peroxidases (lignin peroxidases (EC 1.11.1.14), manganese peroxidases (EC 1.11.1.13), versatile peroxidases (EC 1.11.1.16), dye decolorizing peroxidases (EC 1.11.1.19)), unspecific peroxygenases (EC 1.11.2.1), high-redox potential laccases (EC 1.10.3.2) and enzymes that supply H_2_O_2_ [4], [5]. Different oxidases can be found in the set of enzymes supplying H_2_O_2_, including: i) glyoxal oxidase (EC 1.2.3.15), a copper radical enzyme that works in synergy with lignin and manganese peroxidases [6]; ii) pyranose oxidase (EC 1.1.3.10), a FAD-dependent enzyme with an activity that is also related to the reduction of the quinones produced during lignin combustion [7]; and iii) AAO, which coordinates its oxidative activity with the action of intracellular dehydrogenases, establishing an aromatic alcohol/aldehyde redox cycle that generates a constant supply of H_2_O_2_ for ligninolytic peroxidases and peroxygenases [8], Fig. 1.

AAO activity was first detected in *Trametes versicolor* cultures [10] and since then, it has been found in several other species, including *Fusarium* species [11], [12], *Pleurotus* species [13], [14], [15], [16], [17], *Bjerkandera adusta* [18], *Botrytis cinerea* [19], *Geotrichum candidum dec 1* [20], *Phanerochaete chrysosporium* [21] and *Ustilago maydis* [22]. Initially located on the hyphal surface, the monomeric extracellular AAO acts on lignin-derived molecules, as well as on aromatic fungal metabolites. Additionally, the H_2_O_2_ released generates highly reactive hydroxyl radicals via the Fenton reaction (Fe^2+^ + H_2_O_2_ → OH^•^ + OH^–^ + Fe^3+^). These oxygen species can act as diffusible electron carriers that help to depolymerize lignin structures [8].

AAO has a wide substrate scope and it is capable of oxidizing primary benzyl alcohols with different chemical structures, releasing hydrogen peroxide as byproduct. The enzyme displays its highest activity with electron donors on the aromatic ring like *p*-methoxybenzyl alcohol (anisyl alcohol), the natural substrate of the enzyme and a metabolite present in fungal cultures. In addition, AAO can oxidize unsubstituted benzyl alcohol and halogenated benzyl alcohols with similar reaction rates. Not only does AAO act efficiently on aromatic molecules but it is also very active on aliphatic polyunsaturated alcohols like 2,4-hexadien-1-ol [23]. The substrate palette of alcohols was recently expanded with the conversion of the single unsaturated *trans*-2-hexen-1-nol for the preparation of aromatic chemical *trans*-2-hexenal [24]. As already suggested through the presence of excess stoichiometric molecules of H_2_O_2_ upon alcohol oxidation, AAO also catalyzes the oxidation of aromatic aldehydes into their corresponding acids. The preference for *p*-nitrobenzaldehyde, with an electron withdrawing substituent on the aromatic ring, suggests an analogous reaction mechanism as in the oxidation of alcohols via *gem*-diol species (*i.e.* the hydrated form of an aldehyde containing two hydroxyl groups placed on the same carbon atom) [25].

### 1.2 The AAO from *Pleurotus eryngii* as a part of the GMC superfamily

From a taxonomic point of view, AAO belongs to the GMC (glucose-methanol-choline) superfamily of oxidoreductases, which also includes the flavoenzymes glucose oxidase (EC 1.1.3.4), methanol oxidase (EC 1.1.3.13), choline oxidase (EC 1.1.3.17), cholesterol oxidase (EC 1.1.3.6), cellobiose dehydrogenase (EC 1.1.99.18), pyranose oxidase (EC 1.1.3.10) and 5-hydroxymethylfurfural oxidase (EC 1.1.3.47) [26], [27]. The architecture of GMC oxidoreductases is generally related and it is based on several consensus sequences, such as the conserved ADP-binding ßαß motif in the N-terminal domain, representing a super-secondary structure associated with nucleotide binding (Rossmann fold) that is present in the FAD-binding domain of AAO [28], [29]. The GMC group of oxidoreductases is a large superfamily of quite diverse enzymes. After phylogenetic sequence analysis, several trees were produced with different individual monophyletic clades based on topology, taxonomy and characterized sequence space. Three clades can be found on the reconstructed tree corresponding to the aryl-alcohol oxidase-pyranose dehydrogenase (AAO-PDH) cluster. The AAO-PDH cluster is a relatively uniform cluster that describes a close relationship for both enzymes. It is considered that PDH recently evolved from the AAO after a substrate specificity change. Also the AAO and the aryl-alcohol dehydrogenase are not located in different clades, indicating that small residue changes can shift oxygen specificity on these flavoenzymes [30]. Of the pool of AAOs identified, the enzyme from *P. eryngii* has been most extensively characterized. The first description of AAO activity in *P. eryngii* cultures dates back to 1988 [31], although it was not until the early nineties that the enzyme was purified and it was characterized as a monomeric flavoprotein of 72.6 kDa with around 15% glycosylation [15]. The ensuing cloning of the AAO gene [32] opened the door to its heterologous expression in *Emericella nidulans* [33], as well as in *Escherichia coli* followed by *in vitro* refolding [34].

**Fig. 2:**
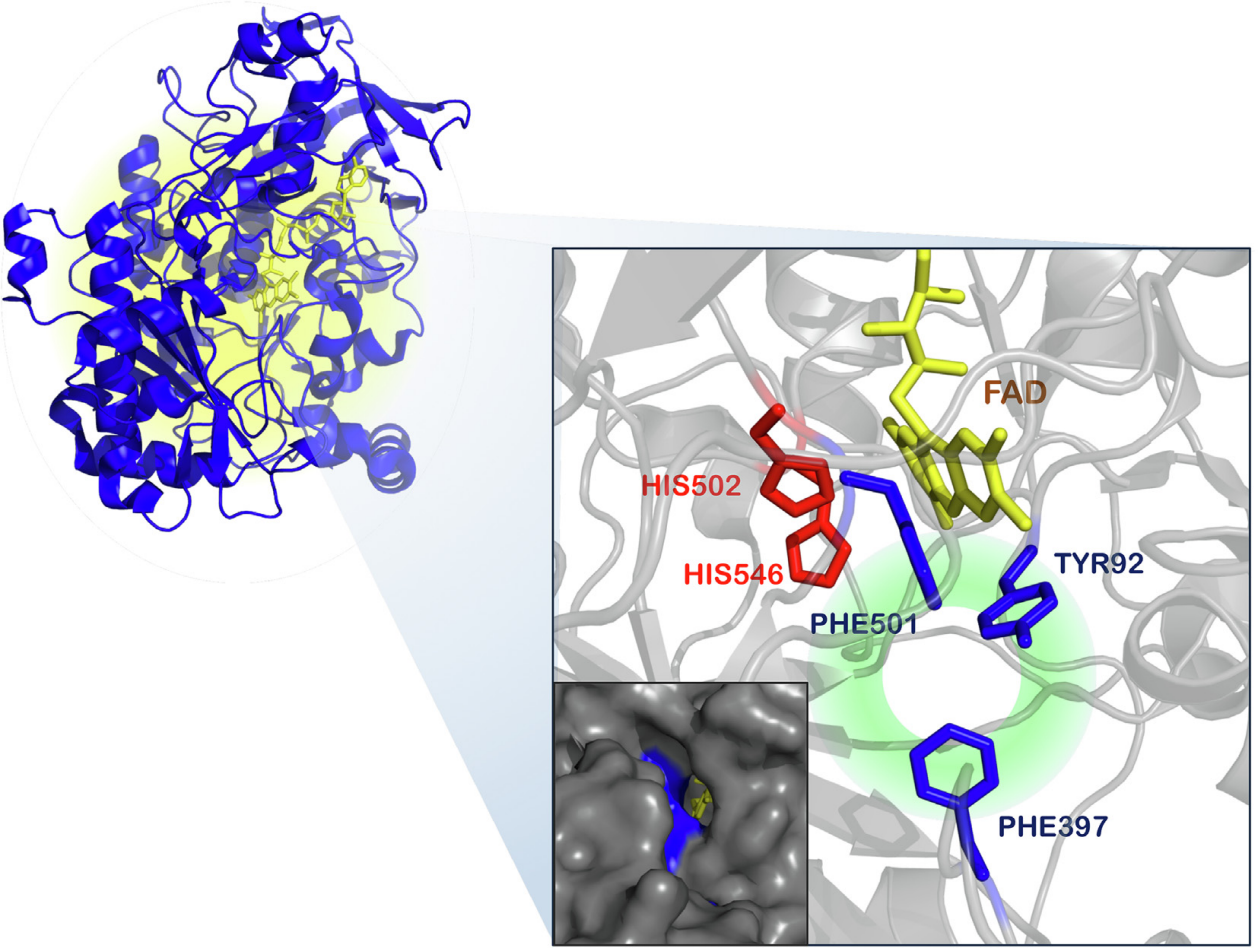
AAO crystal structure. Mapping of the residues forming the aromatic constriction (blue), the catalytic His residues (red) and the FAD (yellow). The inset shows the access channel to the active site (in surface mode). Model based on the PDB structure 3FIM. (For interpretation of the references to colour in this figure legend, the reader is referred to the web version of this article.)

In 2009, the crystallographic structure of recombinant AAO from *P. eryngii* was resolved at a resolution of 2.4 Å upon expression in *E. coli* and *in vitro* refolding (_EC_AAO, PDB entry: 3FIM), Fig. 2. Two main domains were identified in the 566 residues polypeptide, the FAD binding domain and the substrate binding domain. The most interesting topological feature of the protein is the buried active site, where solvent access is strictly limited by an aromatic constriction. Hydrophobic channels that give access to the active site are related to molecular oxygen as substrate. This restricted access to the active cavities is also found in other flavin-dependent oxidases, such as cholesterol oxidase [35] and vanillyl alcohol oxidase [36]. As observed in the crystal, the catalytic cavity contains a non-covalently bound FAD molecule, and the catalytic His502 and His546, while the hydrophobic bottleneck connecting this active site with the solvent is formed by the Tyr92, Phe397 and Phe501 residues [28]. Site-directed mutagenesis experiments at positions Tyr92, Phe501, His502 and His546 have highlighted the importance of those residues for the catalytic activity of the AAO. Aromatic residues should be placed at positions 92 and 501 since mutants Y92F and F501Y maintain activity while variants Y92A and F501A dramatically lose catalytic performance. Likewise, it was shown that His502 and His546 could not be replaced by other residues being their presence essential for catalysis [37].

### 1.3 Catalytic mechanism

**Fig. 3:**
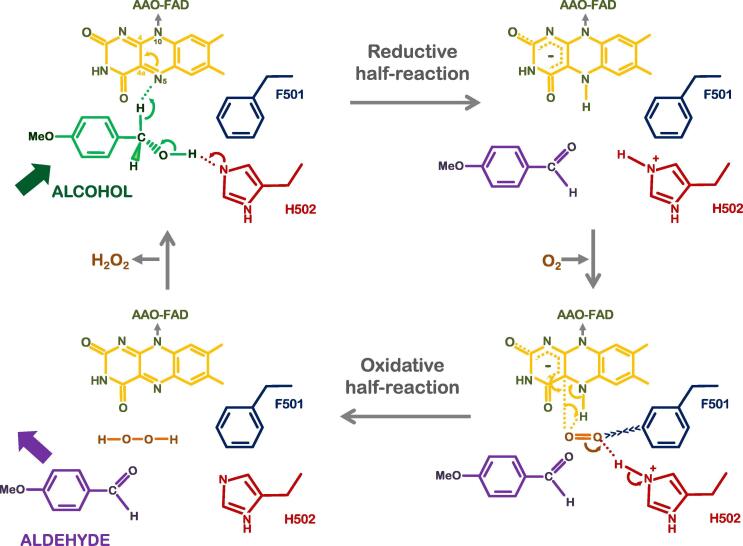
AAO catalytic cycle. Details of the half-reactions catalyzing the oxidation of alcohols into aldehydes.

Adapted from [8].

Insights into the mechanism of action of AAO have been gained through several studies over the past few years, using both experimental and computational approaches [38], [39], [40]. Accordingly, the catalytic cycle of AAO involves dehydrogenative oxidation through two half-reactions, Fig. 3:

(i) a reductive half-reaction where the donor alcohol is two-electron oxidized by FAD, receiving the cofactor of the α-Hs from the alcohol through highly enantioselective hydride transfer to yield an aldehyde product and a reduced flavin.

(ii) the oxidative half-reaction in which molecular oxygen is two-electron reduced by FAD to produce H_2_O_2_ and to leave the cofactor reoxidized.

Alcohol oxidation is the rate limiting step of AAO catalysis, with hydride transfer to the cofactor and proton transfer to the catalytic base. This half-reaction takes place via a concerted non-synchronous bond breaking mechanism. Few structural determinants have been established as key elements in the oxidation driven by AAO. Substrate diffusion studies have shown that Phe397 of the hydrophobic funnel oscillates with the substrate, acting as a gate in the pathway to regulate substrate positioning and product release [41]. With further migration, Tyr92 stabilizes the benzylic ring of the substrate via π-π stacking interactions [42]. The third residue from the aromatic constriction, Phe501, plays an important role in the positioning of O_2_ [43]. Finally, the core of the active site has two histidine residues, consensus elements in the GMC superfamily, with His502 acting as catalytic base and His546 facilitating substrate positioning [44].

### 1.4 Biotechnological and industrial applications

**Fig. 4:**
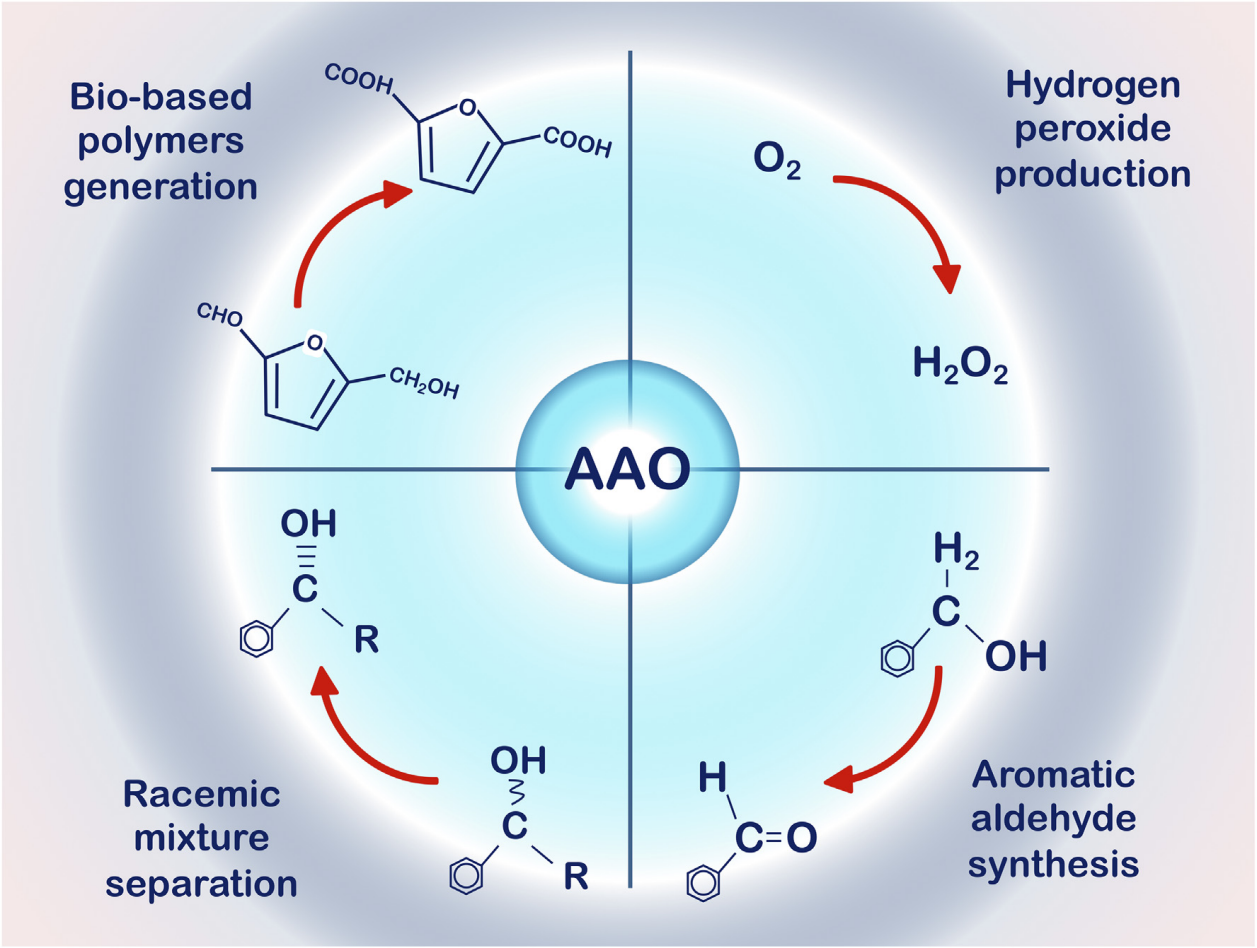
Scheme of the biotechnological applications of AAO.

The activity of AAO on a broad range of alcohols and aldehydes, together with the production of H_2_O_2_ from atmospheric O_2_, has awakened biotechnological interest in relation to several possible industrial applications, Fig. 4. By mimicking its natural role within the ligninolytic consortium, AAO could constitute a part of a self-sufficient synthetic secretome in an adequate industrial host like yeast (specifically “white-rot” yeast) [45], [46]. An engineered microbe that includes the most interesting fungal enzymes could fulfil a number of functions in biorefineries, such as in the production of biofuels and added value chemicals. It is also worth noting that the gradual release of H_2_O_2_ may be appropriate in self-sufficient systems with peroxidases/peroxygenases to ensure efficient cascade reactions. Such systems could be explored through the co-expression of the enzymes or through the creation of chimeric fusion proteins [45], [47]. In the paper industry, the use of laccase combined with AAO for pulp bleaching is another collaborative example [48], although the potential use of AAO as a biocatalyst in organic synthesis should not be underestimated. One characteristic case is the production of flavors and fragrances, such as aromatic aldehydes like benzaldehyde (bitter almond aroma), cinnamaldehyde (cinnamon flavor and aroma) or anisaldehyde (anisic aroma) [49]. However, the resolution of chiral mixtures of alcohols and the catalysis of furfural-derivate cascade reactions are also among the most exciting applications for AAO biotransformations. Indeed, they are an important motivation for the engineering of the enzyme by directed evolution and therefore, they will offer important insights for the following sections.

## 2 Directed evolution of the aryl-alcohol oxidase

**Fig. 5:**
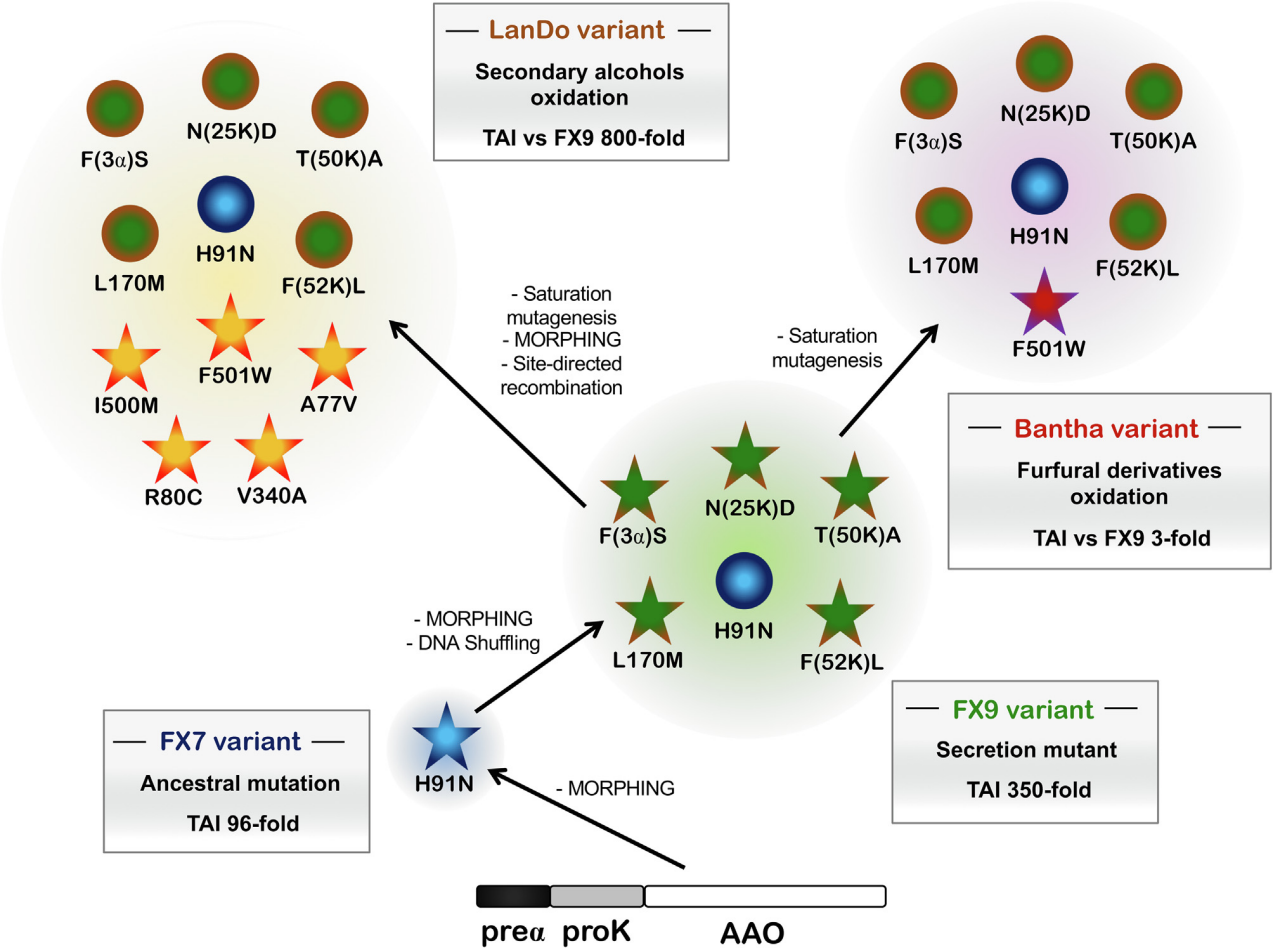
Different routes followed during the directed evolution of AAO. The stars indicate the newly acquired mutations, while the circles depict accumulated substitutions. The TAI (Total Activity Improvements) were determined with *p*-methoxybenzyl alcohol as the substrate for evolution towards secretion, with 1-(*p*-methoxyphenyl)-ethanol as the substrate for evolution for secondary alcohol oxidation and with 5-hydroxymethylfurfural in the evolution for FDCA synthesis.

**Fig. 6:**
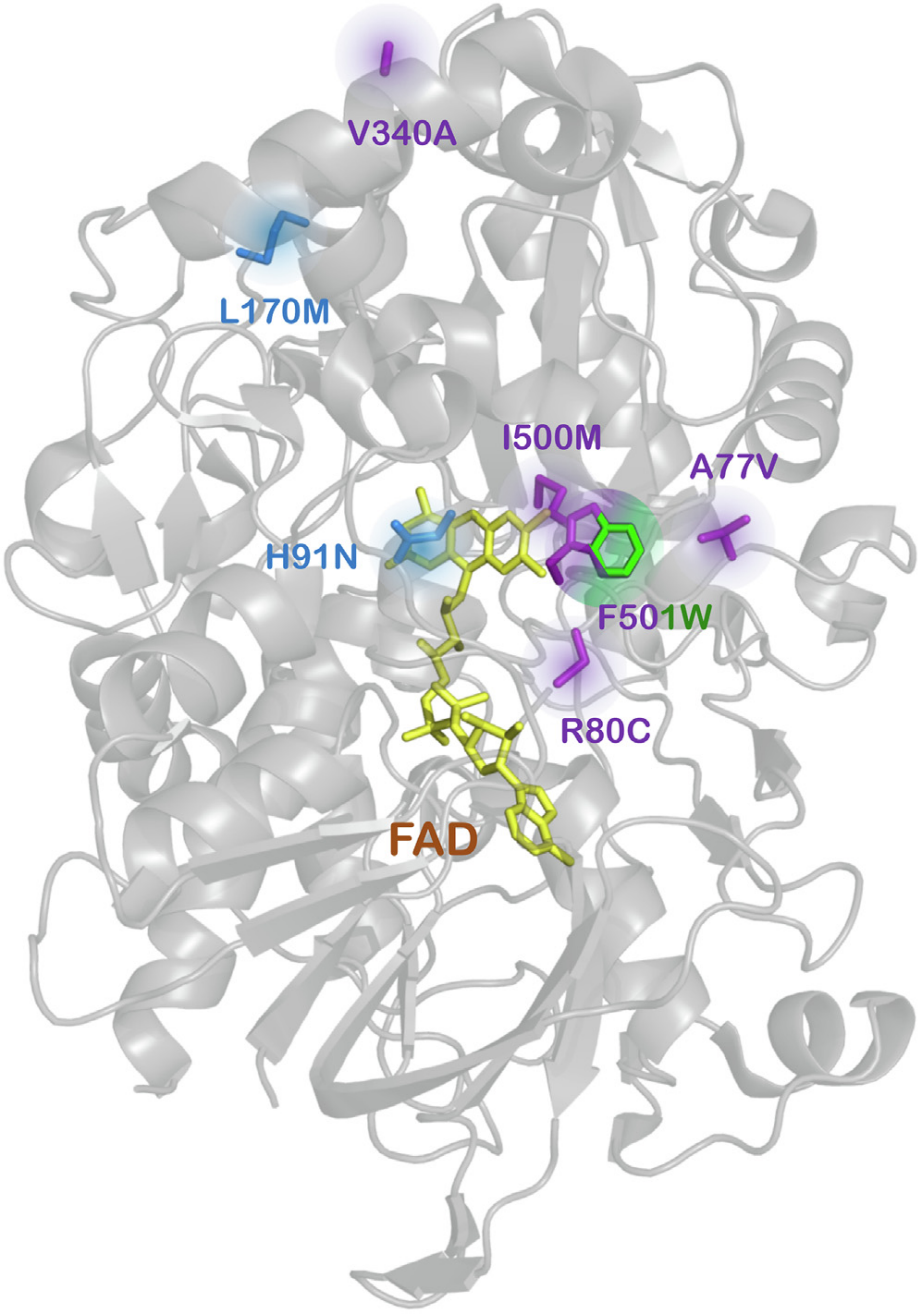
The mutations identified in AAO throughout the directed evolution campaigns. FAD is depicted in yellow and the catalytic histidine residues in red. The mutations corresponding to the evolution of secretion are indicated in blue, and those for the evolution of secondary alcohol oxidation are in purple, while mutations corresponding to furfural derivative oxidations are depicted in green. Substitution F501W, from two evolution campaigns, is depicted in both purple and green. Note that the H91N mutation enhances substrate promiscuity in the FDCA route. (For interpretation of the references to colour in this figure legend, the reader is referred to the web version of this article.)

Until directed evolution was employed to produce AAO variants of biotechnological interest, the enzyme had only been subjected to protein engineering by rational design. These initial site-directed mutagenesis efforts directed towards AAO have been reviewed elsewhere [50] so here, we will describe the pathway our laboratory followed to evolve a set of AAOs with enhanced functional expression [51], [52], [53], increased activity on secondary alcohols [54] and improved performance on furfural derivatives [55], Fig. 5, Fig. 6, Table 1.

**Table 1:** Characterization of the different AAO variants.

Variant	Evolutionary campaign	Mutations	Library creation method	Heterologous host	Secretion level	Total activity improvement(in fold)	*T*_50_ (°C)	Specific activity	Catalytic efficiency*(k*_cat_*/K*_m)_	Reference
preαproK-AAO(parental type)	Functional expression	---	---	*S. cerevisiae*	N.D^1^	1	58.8	N.D^1^	N.D^1^	[51]
FX7	Functional expression	H91N	MORPHING	*S. cerevisiae P. pastoris*	2 mg/L (*S.c*)N.D. (*P.p*)	96^2^	64.3	24 U/mg^3^	1562^3^	[51]
FX9	Functional expression	F[3α]S N[25K]D T[50K]A F[52K]L L170M H91N	DNA Shuffling MORPHING	*S. cerevisiae P. pastoris*	4.5 mg/L (*S.c*)4.3 mg/L (*P.p*)	350^2^	63	34 U/mg^3^	1782^3^	[53]
LanDo	Secondary alcohols oxidation	F[3α]S N[25K]D T[50K]A F[52K]L L170M A77V R80C H91N V340A I500M F501W	CSM MORPHING *Ep-*PCR SDR	*S. cerevisiae*	4.6 mg/L	800^4^	N.D	2.9 U/mg^5^	7.5^5^	[54]
Bantha	Furfural derivatives transformation	F[3α]S N[25K]D T[50K]A F[52K]L L170M H91N F501W	CSM	*S. cerevisiae*	N.D.	3^6^	N.D	N.D	0.76^7^	[55]

1 The first parental type was not purified to homogeneity since there was not enough protein functionally expressed. ^2^ Calculated with reference to parental preαproK-AAO for the oxidation of *p*-methoxybenzyl alcohol. ^3^ With *p*-methoxybenzyl alcohol as substrate. ^4^ Calculated with reference to parental FX9 for the oxidation of 1-(*p*-methoxyphenyl)-ethanol. ^5^ With 1-(*p*-methoxyphenyl)-ethanol as substrate. ^6^ Calculated with reference to parental FX9 for the oxidation of 5-hydroxymethyl furfural. ^7^ With 5-hydroxymethyl furfural as substrate. N.D: No determined. *S.c*: *Saccharomyces cerevisiae*. *P.p: Pichia pastoris*. CSM: Combinatorial Saturation Mutagenesis. *Ep-*PCR: Error prone PCR. SDR: Site-Directed Recombination.

### 2.1 Directed evolution platform

The engineering of AAO implies setting-up an evolutionary platform based around a suitable host for expression that is compatible with directed evolution schemes, together with the development of high-throughput assays to screen activity and sort the mutant variants [51].

i) Chimeric fusion genes to express AAO in yeast

*S. cerevisiae* is a widely used host organism and very suitable to express fungal enzymes for *in vitro* evolution experiments [56]. For years, our laboratory has successfully used the prepro-leader sequence of the mating α-factor of *S. cerevisiae* to drive the secretion and evolution in yeast of ligninases. An alternative approach to achieve adequate secretion of a foreign protein involves using the prepro-leader sequence of the K1 toxin. This leader sequence has been much less intensely explored but it has proven useful to express ß-lactamase [57], [58]. This peptide contains the prepro(δ) sequence, which undergoes similar processing to the prepro-leader of the α-factor, fused to a truncated version of the γ-segment with 3 glycosylation sites. To enhance AAO secretion by yeast, we fused the AAO gene to the α-factor prepro-leader (α-AAO), the K1 toxin prepro-leader (K-AAO), shuffled variants of the preα sequence with the K1 γ-segment (preαproK-AAO) and the prepro(δ) sequence with the proα sequence (preKproα-AAO). As all the fusions were successfully processed and exported by *S. cerevisiae*, the engineering of these chimeric leaders provided novel strategies to secrete foreign proteins so that they may be tested on other eukaryotic genes.

ii) Dual high-throughput screening (HTS) for AAO activity

AAO activity implies an increase in H_2_O_2_ concentration in the yeast supernatant. To accurately quantify the AAO activity during screening, we used the FOX assay, a very sensitive method based on the Fenton reaction [59]. After oxidation by H_2_O_2_, Fe^3+^ reacts with xylenol orange to form a blue-purple complex. The Fe^2+^ oxidation step can be propagated by the addition of sorbitol, enhancing the Fe^3+^ concentration and hence, the sensitivity of the assay. When tested against *S. cerevisiae* supernatants, the FOX signal was linear and stable, without noticeable interference and with a limit of sensitivity of ~ 0.4 µM in the presence of sorbitol. This assay was complemented by the classic indirect method that is based on coupling H_2_O_2_ production to the activity of horseradish peroxidase (HRP, EC 1.11.1.7) using ABTS as a reporter of activity [51], [52]. Given that the FOX/HRP-ABTS dual HTS assay can be used to explore libraries regardless of the substrate oxidized, it represents a ‘*blank checḱ* for directed AAO evolution, opening up as many pathways of development as needed.

iii) Directed AAO evolution for secretion in *S. cerevisiae* and *Pichia pastoris*

The preαproK-AAO construct was selected as the departure point for directed evolution towards improved expression as its signal preproleader guaranteed measurable activity in yeast supernatants, avoiding the inefficient pro-leader processing by the STE13 protease (proK segment only depends on KEX2 activity). A random mutagenic library on the whole AAO gene was constructed along with focused mutagenesis and recombination by MORPHING, a method where random mutations and recombination events are introduced in determined fragments of the protein leaving the rest of the structure intact [60]. This library creation method enables the mutational load to be directed towards specific areas of the protein, while maintaining the remaining residues unchanged. While several variants presented up to 5-fold improvements in total activity compared to the parental preαproK-AAO, the MORPHING variant FX7 showed a striking 96-fold enhancement in activity, thanks specifically to the consensus/ancestral substitution H91N, Fig. 5, Fig. 6. This mutation occurs at the site of contact with the *si*-side of the FAD cofactor in the catalytic pocket. With secretion levels of 2 mg/L, FX7 was correctly processed and presented similar kinetics to _EC_AAO. Significantly, FX7 was heavily glycosylated (~50%), which possibly stabilizes it. This mutant not only displayed broad pH stability but also, a *T*_50_ value -the temperature at which 50% of the initial activity is retained after 10 min incubation- 11 °C higher than that of _EC_AAO. Later, FX7 was *in vivo* shuffled with other representative variants of the evolutionary campaign [53], giving rise to FX8, which had been subject to at least two recombination events, in which the T50A mutation in the proK segment, the L170M mutation at the surface of the protein and the ancestral H91N were brought together, producing a 2.5-fold improvement in total activity. Secretion was also improved by directing the mutational load to the signal peptide. The ultimate secretion variant, the FX9 mutant, acquired the F[3α]S substitution in the preα segment, in addition to the N[25K]D and F[52K]L mutations in the proK segment. Together with the T[50K]A, L170M and H91N mutations, these modifications increased secretion to up to 4.5 mg/L, giving a total 350-fold improvement over the parental preαproK-AAO. Finally, the FX9 variant was successfully transferred to the industrial host *P. pastoris* for overproduction in a bioreactor, producing 25 mg/L without any optimization of the bioreactor engineering process. This initial level of production demonstrates the potential value of AAO to be developed as a true industrial biocatalyst.

### 2.2 Directed evolution of a secondary benzyl-alcohol oxidase

Chiral chemistry continuously represents a challenge in drug development and indeed, enantiopure building blocks are in strong demand to obtain drugs with particular biological activities [61], [62], [63]. AAO is a promising candidate for the enantioselective oxidation of chiral benzyl alcohols. As mentioned above, the mechanism used by AAO for alcohol oxidation involves hydride abstraction from the Cα position of the alcohol in conjunction with proton transfer to the catalytic base of the enzyme. The stereoselectivity of hydride transfer has been studied extensively [40]. In particular, the relative positions at the active site of the *p*-methoxybenzyl alcohol, the FAD cofactor and the catalytic His502 were estimated by computational ligand diffusion simulations (PELE: protein landscape exploration). Unfortunately, the activity towards secondary 1-(*p*-methoxyphenyl) ethanol appears to be residual, with an apparent efficiency orders of magnitude lower than that towards primary alcohols. On the grounds that a wider space in the restricted active site would avoid steric hindrance and better accommodate a bulkier secondary substrate, the F501A variant originally prepared in 2006 was created to remove the side chain of the aromatic residue. As a result, activity towards primary alcohols was reduced while the relative activity on secondary substrates improved [40]. More recent computational studies indicated both Ile500 and Phe501 represent the main steric obstacles in the accommodation of bulkier 1-(*p*-methoxyphenyl)-ethanol [64].

Along similar lines, we constructed the I500A, F501A and I500A-F501A variants of the FX9 secretion mutant. Unfortunately, no activity on the secondary alcohol was detected upon microfermentation of these mutants. In the hope of finding a better amino acid configuration at those key positions, a combinatorial saturation mutagenesis library was prepared targeting positions Ile500 and Phe501. Surprisingly, the 15G12 variant containing the bulkier I500M-F501W substitutions was the best performer, with a 160-fold improvement in oxidizing 1-(*p*-methoxyphenyl)-ethanol. The modified activity profile of the 15G12 variant led to a loss of most of its activity on AAO’s natural substrate, *p*-methoxybenzyl alcohol, yet it was able to oxidize the secondary substrates *p*-fluoro-α-methylbenzyl alcohol and 1-phenylethanol [54]. Once the residual activity on the secondary alcohol was unlocked, we widened the structural search for enhancing mutations by MORPHING. Three mutagenic blocks were addressed, involving the access channel and catalytic pocket of the 15G12 variant, the MA (Leu48-Thr100), MB (Leu310-Ile417) and MC (Glu490-Gln566) blocks. This approach was particularly successful in uncovering the importance of the MA block, involving the access channel and FAD-binding domain, and with around 70% of the selected mutations situated in a domain separated by no more than 14 residues: I76V, A77V, R80C, M83I and V90A. Further rounds of mutagenic DNA shuffling yielded improvements from 220- to 410-fold relative to the FX9 parental type.

**Fig. 7:**
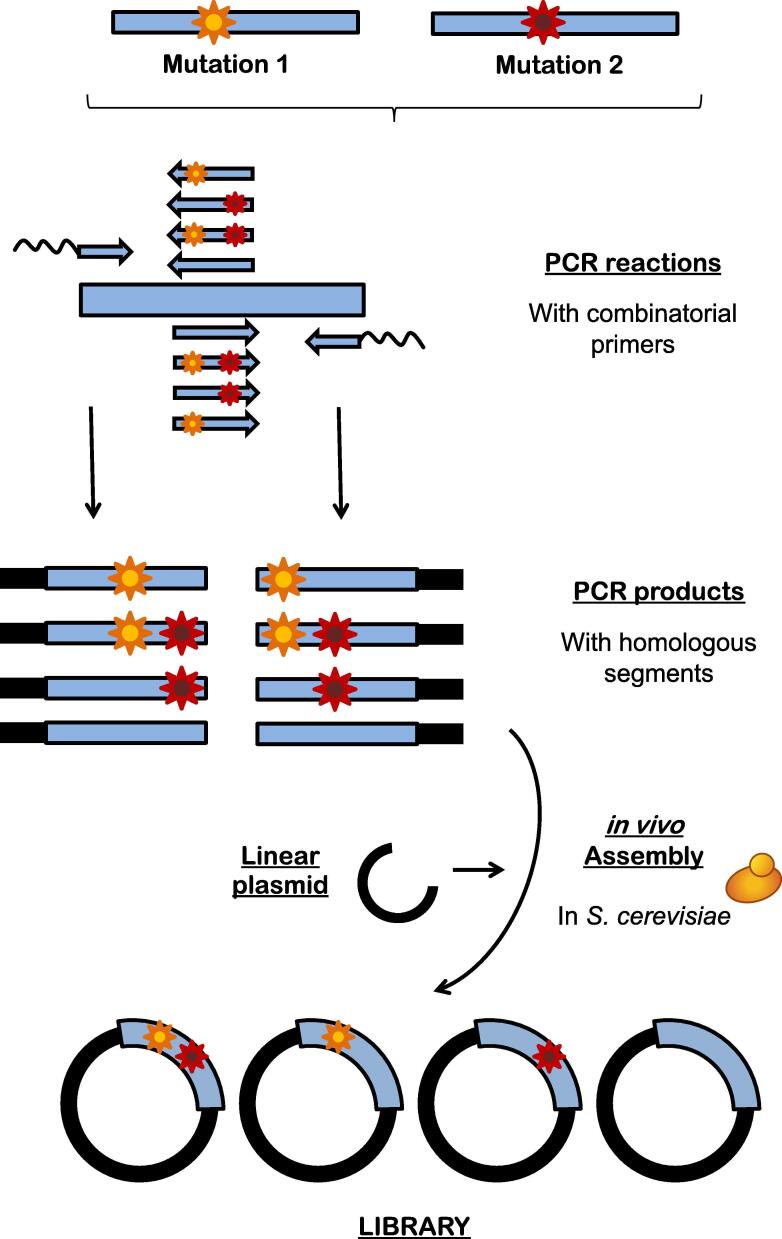
The site directed recombination method. PCR fragments are assembled containing 50% mutated and 50% wild type residues at the selected codons. After that, the fragments are cloned into yeast with the help of homologous overlapping regions. This way, mutations/reversions at the different positions can be studied in a combinatorial display.

To explore the possible epistatic effects of the panel of substitutions, a site-directed recombination library was constructed with 10 selected mutations. The site-directed recombination approach generates genetic diversity when the selected mutations and their correspondent reversions are studied in a combinatorial manner [65], Fig. 7. This strategy led to the identification of the LanDo variant, which carried the A77V-R80C-V340A-I500M-F501W mutations along with the secretion mutations of FX9, and that displayed an 800-fold improvement in the oxidation of secondary alcohols, Fig. 5, Fig. 6. Indeed, LanDo showed a striking increase of three orders of magnitude in terms of its catalytic efficiency for the secondary alcohol used in the screening relative to _EC_AAO. In addition, the initial turnover rates for *p*-fluoro-α-methylbenzyl alcohol and 1-phenylethanol increased 20- and 100-fold, and LanDo unlocked its activity towards 1-phenylpropanol, which was undetectable with the native enzyme. The recovery of activity on the primary *p*-methoxybenzyl alcohol was also noteworthy, a trait almost totally lost in the 15G12 variant. The essential characteristic to resolve chiral mixtures, over and above the total activity with secondary alcohols, is a high preference for one of the alcohol enantiomers. To determine the enantioselectivity of LanDo, transformation of the racemic 1-(*p*-methoxyphenyl)-ethanol was studied by chiral-HPLC. The enantiomeric excess when the reaction was completed was greater than 99% after a 90 min reaction, corresponding to the R enantiomer. This result implies that natural selectivity of the AAO was maintained throughout the evolution process.

A computational interpretation of these results was carried out by PELE. At the atomic level, R80C in particular created an empty space on top of the FAD. In addition, since Arg80 interacts with the backbone of Trp501, the introduction of the Cys80 mutation allows Trp501 to form a hydrogen bond with Val77. In summary, minor conformational changes were responsible for remarkable improvements in catalytic performance, making this evolved AAO variant a promising biocatalyst for the resolution of racemic mixtures of benzyl alcohols [54].

### 2.3 Directed evolution for the transformation of furfural derivatives

**Fig. 8:**
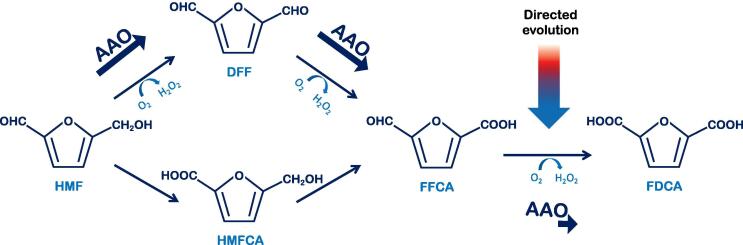
Oxidative pathways for the transformation of HMF into FDCA.

Greener renewable technologies can be developed for the efficient production of added value molecules and platform chemicals from biomass feedstocks. One of the bio-derived chemicals with the biggest biotechnological potential is 5-hydroxymethylfurfural (HMF), which can easily be obtained from biomass as a product of sugar dehydration [66]. Three consecutive oxidations transform HMF into furan-2,5-dicarboxylic acid (FDCA), a promising building block that can be used in the polymer industry. FDCA can be enzymatically prepared from HMF through sequential two e^-^ oxidation of two furanic intermediates, 2,5-diformylfuran (DFF) and 5-formyl-2-furancarboxylic acid (FFCA). This biocatalytic conversion has mainly been studied for two oxidases from the GMC superfamily, AAO from *Pleurotus eryngii* and the bacterial 5-hydroxymethylfurfural oxidase (HMFO) from *Methylovorus sp*. As AAO is apparently not active on FFCA, full conversion into FDCA was achieved by coupling the oxidase with the UPO from *Agrocybe aegerita* in a self-sufficient enzymatic cascade. This system allows AAO to transform HMF into FFCA, producing the H_2_O_2_ needed by the UPO as a co-substrate for the oxidation of the formyl group of FFCA to finally obtain FDCA [67]. By contrast, three different AAOs only moderately transformed FFCA directly into FDCA, the native enzymes from *Pleurotus eryngii*, *Pleurotus ostreatus* and *Bjerkandera adusta*, yet failing to achieve FDCA production when HMF was the initial substrate [68]. Exploring the hidden abilities of AAO in this challenging cascade is definitely of interest, Fig. 8. HMFO presents a catalytic profile similar to AAO and apart from furfural derivatives, this flavoenzyme mainly acts on primary aromatic alcohols and conjugated aliphatic systems like 2,4-hexadien-1-ol. Similarly, HMFO cannot oxidize secondary alcohols and it is active on hydrated aldehydes. Taking into account these common characteristics, it is interesting that the sequence similarity between both oxidases is remarkably low (<30%) and that their natural roles also clearly diverge, particularly considering HMFO is an intracellular enzyme [69]. In the case of HMFO, FFCA oxidation has been explored by producing a double V367R-W466F variant with catalytic efficiency for FFCA enhanced by three orders of magnitude. However, the *k*_cat_ of the new variant was one order of magnitude lower than that of the native enzyme with HMF [70].

Given that all reaction intermediates in the FDCA route should be accepted as substrates, the first plan of action for the evolution of AAO was the benchmarking of its variants in order to establish their capacity to oxidize both the initial HMF and the final FFCA intermediate [53], [54]. As expected, no activity was detected with FFCA in the HTS format, whereas the volumetric activity of FX9 on HMF (0.3 U/L) was more than 30-fold higher than the activity of the 15G12 variant. Indeed, it was evident that positions 500 and 501 were determinant for activity on the furfural derivate, as was reported in the case of HMFO [70]. The strategy followed involved combinatorial saturation mutagenesis (CSM) of these positions and other structurally related residues (after alignment with HMFO) to find a better biocatalyst for the reaction than FX9. After several CSM experiments, only one combination of amino acids improved HMF oxidation (3-fold), the Bantha mutant with the F501W substitution and a 60-fold enhancement in *k*_cat_, Fig. 5, Fig. 6. With improved catalytic efficiency for HMF, we wondered whether or not the new variant could complete the whole cascade reaction from HMF to FDCA, with enhanced activity on FFCA. The production of FDCA from HMF and FFCA by Bantha or FX9 was analyzed by HPLC, and compared with _EC_AAO. After a 24 h incubation, the transformation of FFCA by Bantha was 7-fold more efficient than that of _EC_AAO, while the FX9 variant also mediated the whole pathway, albeit less strongly. Moreover, after both enzymes transformed HMF fully to FFCA in a 48 h reaction, the subsequent oxidation into FDCA by Bantha was 6 times more efficient. Thus, for the first time AAO was seen to be capable of completing the entire three consecutive oxidation cascade [55].

The AAO variant Bantha carries two substitutions that are paramount for its completion of the FDCA route: H91N and F501W. The back-to-consensus ancestor mutation H91N was responsible for broadening promiscuity towards FFCA, as this activity was already observed in the FX9 variant that carries the H91N mutation. Conversely, the F501W substitution was behind the overall improved activity from HMF, helping to situate O_2_ adequately for better reactivity. In fact, a Trp residue can be found at the homologous position in other AAOs, such as the enzyme from *Bjerkandera adusta* [43]. It is worth noting that HMFO also has a Trp in the corresponding position (W466), as well as Asn at position 102, equivalent to the ancestral/consensus H91N mutation acquired in the evolutionary campaign for functional expression [51]. Indeed, this enzyme did complete the whole FDCA route in its native form. The effect of the mutations in Bantha was rationalized by *in silico* PELE simulations. In contrast to the wild-type, correct diffusion of the FFCA substrate into Bantha’s active site was observed. In summary, far from being an optimal process, the substitutions discovered during the evolution of AAO add to the enzymatic toolbox for FDCA preparation [55].

## 3 Conclusions

This review has showcased the last developments in the engineering of AAO for industrial applications. Early efforts elucidated the most characteristic structural determinants of AAO, paving the way for directed evolution to further push its biotechnological potential. Specifically, directed evolution allowed AAO to be functionally expressed in yeast, whereas a collection of new AAOs variants have been generated with modified activities towards substrates of interest, such as secondary aryl-alcohols and furfural derivatives. At this point, directed evolution is an essential part of the development of AAO as a future industrial biocatalyst, and the prospects include its use as part of the enzymatic cascade for in situ H_2_O_2_ production, or for the design of chimeric fusion proteins with peroxygenases for multistep reactions.

## Declaration of Competing Interest

The authors declare that they have no known competing financial interests or personal relationships that could have appeared to influence the work reported in this paper.
